# News reporting of suicide in nurses: A content analysis study

**DOI:** 10.1111/inm.13057

**Published:** 2022-08-25

**Authors:** Samantha Groves, Monica Hawley, Karen Moya Lascelles, Keith Hawton

**Affiliations:** ^1^ Oxford Health NHS Foundation Trust Warneford Hospital Oxford UK; ^2^ Samaritans The Upper Mill Ewell UK; ^3^ Centre for Suicide Research, Department of Psychiatry University of Oxford, Warneford Hospital Oxford UK

**Keywords:** COVID‐19, news reporting, nurses, students, nursing, suicide

## Abstract

Media impact on suicide is well‐established. Groups at heightened risk of suicide, such as nurses, may be particularly influenced by poor news reporting. This study aimed to examine UK newspaper reporting of suicide of nurses and student nurses, including during the COVID‐19 pandemic. Print and online newspaper reports about suicide in nurses (including students) published in the UK between January 2018 and August 2021 were obtained and data extracted for analysis in collaboration with Samaritans' media advisory team. Content and quality of newspaper reports were examined using a content analysis approach. The study was compliant with the STROBE checklist. Nurse or student nurse suicides were reported in 134 articles, including 50 individual suicides. Most articles were acceptable against Samaritans' media guidelines. However, common problems included absence of signposting to support organizations and lack of suicide prevention messages. A minority of articles included methods of suicide within article headlines (18, 13.4%) and sensationalist or romanticizing language (14, 10.7%). Most contained occupation‐related content. Many named the individual's specific hospital or university and a substantial proportion included occupation‐related images. Working on the frontline was the most reported link between COVID‐19 and nurse suicide. While reporting on suicide among nurses and students was largely acceptable, quality of reporting was variable. Occupation was often discussed, and most articles published during COVID‐19 linked suicide to the pandemic. The research findings can help shape guidance on reporting of suicide in specific professions and occupations, including nursing, to encourage responsible reporting and reduce inadvertent promotion of suicide.

## BACKGROUND

More than one in every 100 deaths worldwide are the result of suicide and prevention of suicide is a global priority (World Health Organization 2021). Reporting of suicide in the media, including in news reports, has been highlighted as having a role in suicide prevention and forms part of national suicide prevention strategy in the United Kingdom (UK) (Department of Health 2012) and worldwide (World Health Organization 2014).

Suicide and self‐harm can be influenced by the reporting of suicide in the media (Pirkis *et al*. 2018), particularly in vulnerable populations, such as children and young adults, or individuals with a history of depression (Cheng *et al*. 2007; Gould *et al*. 2014). When reports of suicide are highly visible (such as front page news or clearly stating suicide in the headline), dramatic, or are focused on celebrities, this impact may be increased (Niederkrotenthaler *et al*. 2010; Sinyor *et al*. 2018). For example, a meta‐analysis showed rates of suicide increased after a celebrity suicide, with up to a 30% increase when method of suicide was reported (Niederkrotenthaler *et al*. 2020).

In response to the influence of reporting of suicide, several organizations, including the World Health Organization (2017) and the UK suicide prevention charity Samaritans (2020, 2022), have developed guidance for media reporting on suicide. Samaritans' guidance is targeted to media professionals and includes recommendations, such as the need to avoid reporting suicide method, dramatic headlines, and sensational or dramatic language or images. Guidance also suggests that signposting to sources of support should be included (Samaritans 2020). Further, specialized guidance for reporting during the COVID‐19 pandemic (Hawton *et al*. 2021a; Reidenberg & Niederkrotenthaler 2020) and on suicides of high‐profile individuals (Samaritans 2020) has also been developed. Newspaper compliance with guidelines for reporting suicide has been shown to be variable in the UK, both before (Marzano *et al*. 2018; Pitman & Stevenson 2015) and during the pandemic (Marzano *et al*. 2022), including in health professionals (Lynn‐Green *et al*. 2021).

The quality of media reporting on suicide is of particular importance when considering groups of individuals at already heightened risk. Irresponsible reporting may contribute to contagion of suicidal behaviour through imitation (Pirkis *et al*. 2018); however, sensitive reporting may facilitate a protective effect (Till *et al*. 2018). Nurses are at increased risk of suicide compared with the general population, with female nurses being particularly vulnerable (e.g. Alderson *et al*. 2015; Davis *et al*. 2021; Windsor‐Shellard & Gunnell 2019). Concerns surrounding suicides of nurses have been heightened during the COVID‐19 pandemic (e.g., Rahman & Plummer 2020; Ross 2020). Preliminary research has fortunately indicated that the suicide rate among the general population in the UK has not increased since the onset of the pandemic (e.g. Appleby 2021). However, the impact of COVID‐19 on the suicide rate of health workers, including nurses, has not yet been quantified. International research has measured suicide and self‐harm ideation among nurses working during the pandemic (e.g. Ariapooran & Amirimanesh 2020; Greenberg *et al*. 2021; Hong *et al*. 2021; Lixia *et al*. 2022; Robles *et al*. 2020; Xu et al., 2021). Prevalence of ideation ranged from 1.5% to 37.5% across studies. However, it is not known whether this represents a change in ideation prevalence compared with prior to the pandemic. Occupational and COVID‐19‐related factors, such as infection of family members and perceived workplace support have been shown to be associated with suicide ideation (Hong *et al*. 2021). However, the quality of evidence related to the impact of the pandemic on suicidal behaviours among healthcare professionals has so far been assessed to be largely poor (Eyles *et al*. 2021).

Recent research has shown that news reporting of suicide in relation to COVID‐19 appears to be skewed towards frontline healthcare staff, such as nurses (Marzano *et al*. 2022). The reporting of suicide among student nurses is also of importance, given that suicide in young people is particularly likely to be influenced by news reporting on suicide (Gould *et al*. 2014). In addition, extensive coverage of nurse and nursing student suicide may prevent prospective students entering training, or discourage current nurses from staying in the profession, contributing to the current nursing shortage. Understaffing due to a lack of nurses has been shown to increase burnout (Baker *et al*. 2019; Lasater *et al*. 2021), which is associated with increased risk of suicide ideation among nurses (Chin *et al*. 2019; Kelsey *et al*. 2021).

We have investigated UK news reporting of suicide among nurses and associated students, including a focus on coverage during the COVID‐19 pandemic, with the aim of determining the nature and quality of the reports.

## METHODS

### Data identification and extraction

The present study examines UK newspaper reporting of suicide. Therefore, as Samaritans' reporting guidelines are the principal recommendations in the UK, these were used as a framework for this study. Samaritans routinely collects newspaper (print and online) reports published in the UK that focus on suicidal behaviours (Fraser *et al*. 2017). Reports are provided by a media monitoring company to Samaritans for analysis by trained staff. The staff extract basic characteristics of the reports (e.g., headline, article focus) and assess their compliance against Samaritans' guidelines, focusing on article headline, content and images. Articles are then assessed as ‘positive’, ‘neutral’, or ‘negative’ against Samaritans' guidelines. Since 2018, Samaritans has recorded the occupation of the individual who has died by suicide covered by each article. In response to the pandemic, Samaritans collected further data on reports using an adapted version of the ‘Classification of COVID‐19 related factors involved in self‐harm’ (Hawton *et al*. 2021b) to explore links within articles about the pandemic and suicide.

Reports describing suicidal behaviours among nurses and associated students were extracted and primary data analysis described above (as used by Marzano *et al*. 2018; Marzano *et al*. 2022) shared with the research team (MH is both a member of Samaritans' media advisory team and the present research team). To facilitate further analysis, links to online articles were provided by Samaritans, and archives were manually searched to identify articles published in print (two print articles and one online article were unavailable for secondary analysis).

### Data analysis

A content analysis approach (as employed by Marzano *et al*. 2018) was used for data analysis. This approach has demonstrable utility for analysing reporting of suicide within the UK media (Marzano *et al*. 2018; Marzano *et al*. 2022). A standardized coding framework was developed by the research team, based on Samaritans' primary analyses (including the overall assessment rating) alongside secondary analyses. The latter included codes related to occupation‐based features of each article; for example, whether a hospital or university was named within the text, inclusion of occupation‐related images, and citation of occupation‐related issues within articles (see File S1 for data extraction form based on this coding template).

Each article was double coded by a member of the research team (SG) to confirm Samaritans' primary coding, and secondary analyses were completed. Coding was iteratively conducted, (consisting of deductive and inductive coding) until each code was assessed across all articles. Where required, two members of the research team (SG, MH) discussed discrepancies in coding to achieve consensus. The incidence of each code was then counted. Data are presented as frequencies and percentages. Analyses are presented for each article published, and also for individual stories (which may have been covered by multiple articles).

### Ethical considerations

Ethical review was not required for this study. Contents of reports have been anonymized.

## RESULTS

**FIG. 1:**
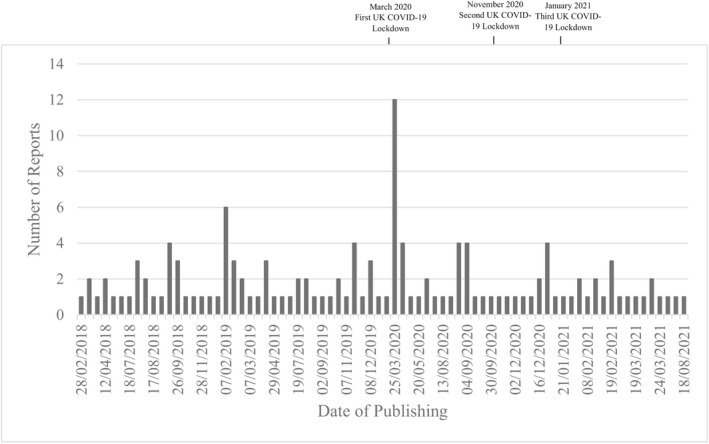
Coverage of newspaper reports of nurse and nursing student suicides (28/02/2018–18/08/21).

From 1 January 2018 to 31 August 2021, 134 articles reporting suicide in nurses and nursing students were identified by the Samaritans' media monitoring service. Full data were available for all but three articles (two print and one online article). Where data were not available for secondary analyses, the denominator is denoted. A mean of three articles per month were published, although in 10 months none were published. In March 2020, there was a peak in reporting following the death of two nurses at the beginning of the COVID‐19 pandemic. A small increase in reporting was also observed following the suicides of two nursing students in February 2019 (Fig. 1). Most articles appeared in tabloid newspapers (68, 50.7%) or local and regional papers (50, 37.3%). A minority of reports appeared in broadsheet (11, 8.2%), broadcast (4, 3.0%), or trade newspapers (1, 0.7%). A summary of article characteristics is displayed in Table 1. The majority of reports were published in online format (81, 60.4%). Regarding article visibility, 21.6% (*N* = 11/51) of print newspapers featured the article on the front page. Of online articles displaying metric data (19/80, 23.7%), articles were shared from seven to over 76 000 times, with 31.3% (*N* = 25/80) of online articles including comments from the public.

**TABLE 1:** Summary of article characteristics

	Non‐COVID	COVID‐related
Total	108 (80.6%)	26 (19.4%)
Newspaper type (*N* = 134)		
Broadsheet	9 (8.3%)	2 (7.7%)
Tabloid	48 (44.4%)	20 (76.9%)
Regional	47 (43.5%)	3 (11.5%)
Broadcast	3 (2.8%)	1 (3.8%)
Trade	1 (0.9%)	0 (0%)
Format (*N* = 134)		
Print	46 (42.6%)	7 (26.9%)
Online	62 (57.4%)	19 (73.1%)
Quality assessment		
Headline (*N* = 134)	Neutral: 94 (87.0%)	Neutral: 22 (84.6%)
	Negative: 14 (13.0%)	Negative: 4 (15.4%)
Images (of articles with images, *N* = 116)	Neutral: 92/94 (97.9%)	Neutral: 22 (100%)
	Negative: 2/94 (2.1%)	Negative: 0 (0%)
Overall (*N* = 134)	Neutral: 108 (100%)	Neutral: 26 (100%)
Reporting problems		
Problem with language (*N* = 131)	4 (3.8%)	10 (38.5%)
Included prevention messages (*N* = 131)	14 (13.3%)	2 (7.7%)
Included signposting (*N* = 134)	55 (50.9%)	17 (65.4%)

### Individual stories of suicide in nurses and nursing students

Of all articles published, 131 (97.8%) were stories about 50 individual suicides (33/50, 66.0% females and 17/50, 34.0% males). Of these, 28 deaths were included in only one newspaper article, 14 in two‐to‐four articles, eight in five or more articles, with one story being covered by 13 articles. Nearly, all deaths were individual suicides (48/50, 96.0%), with two being murder‐suicides (4.0%). Stories were mainly about current nurses (38/50, 76.0%), with a few about former nurses (3/50, 6.0%) and nursing students (9/50, 18.0%). Method of suicide was reported in 74.0% (*N* = 37/50) of stories, with the most common methods reported being hanging (17/37, 45.9%) and overdose (11/37, 29.7%). Three articles (2.2%) discussed multiple nurses who had died by suicide, without a specific focus on a single suicide event. Nine suicide deaths in nurses (18.0%) were reported during the COVID‐19 pandemic (March 2020–August 2021).

### Article headlines

Almost two‐thirds of articles clearly mentioned suicide within the headline (88, 65.7%), with the majority explicitly stating that a death was of a nurse or nursing student (110, 82.1%). A considerable proportion of headlines referenced occupation‐related issues (44, 32.8%), such as work‐based stress and pressure, or job loss. Of articles published during COVID‐19 (*N* = 26), the pandemic was referenced in 18 (69.2%). Headlines were deemed to be largely acceptable according to Samaritans' assessment, with all articles of negative rating (18, 13.4%) including method of suicide within the headline. The stigmatizing terminology of ‘*commit suicide’* was used in two article headlines. Headlines which suggested contributory factors most commonly included citing occupation‐related issues, mental health difficulties, problems with mental health support, traumatic events, and fear of spreading COVID‐19.

### Article text

Half of all articles primarily focused on coroners' inquests into deaths (69, 51.5%), with a quarter focusing on a suicide incident (31, 23.1%), or discussion of a suicide story more broadly (34, 25.4%). Most articles which stated the region where the suicide occurred were about deaths in the UK (121/130, 93.1%), with six articles covering a suicide of a nurse in Italy, and single articles about single suicides in Australia, Ireland, and the United States. Over half of articles made reference to the individuals who died by suicide having past or current mental health difficulties (78/131, 59.5%), with a smaller proportion reporting a history of suicide attempts or self‐harm (22/131, 16.8%). Prior traumatic experiences (27/131, 20.6%), physical health conditions (including COVID infection; 21/131, 16.0%), substance use (6/131, 4.6%), and neurodevelopmental disorders (1/131, 0.8%) were also reported as relevant factors within articles. Most articles contained quotes from individuals bereaved by the suicide, such as family or friends (93/131, 71.0%). However, the source of these quotes varied. For example, quotes taken from statements made at coroner's courts, quotes taken from social media, or quotes provided specifically for an article.

The workplace or university of the individual who died by suicide was named in over two‐thirds of articles (90/131, 68.7%), with 34.4% (N = 45/131) of these providing a quote from a representative of the organization. Colleagues of the deceased were named in over one‐in‐ten articles (16/131, 12.2%) and one report named a previous patient of a nurse. Healthcare professionals who had provided mental healthcare support to the individual who died by suicide were named quite often (22/131, 16.8%). Eleven articles explicitly linked the individual who had died by suicide to other healthcare professionals who had died by suicide, and 12 articles included a general discussion on clustering of suicides in healthcare professionals. Many reports discussed workplace issues as contributing to the suicide of a nurse (62/131, 47.3%), including several inferring lack of support within the workplace (23/131, 17.6%). Sources of information about proposed contributory factors differed by article, including press speculation, coroners' narratives, and quotes from those bereaved.

Twenty‐six articles (19.4%) linked the suicide of a nurse to the COVID‐19 pandemic. Of these, the majority were assessed to include a strong and direct link (16/26, 61.5%). Using the *‘Classification of COVID‐19 related factors involved in self‐harm*’ (Hawton *et al*. 2021b), the most frequently reported links were working on the frontline (22/26, 84.6%), fear of infecting others (9/26, 34.6%), worsening of mental health (5/26, 19.2%), and a combined classification of specific causes of isolation, including separation from friends and family (5/26, 19.2%). Disruption of normal routine (4/26, 15.4%), fear of infection (2/26, 7.7%), and alcohol or drugs (1/26, 3.8%) were also cited as contributory factors. However, proposed contributory factors were again based on speculation by the press, or individuals quoted within the report. Other links within the classification, such as bereavement due to COVID‐19, housing or financial issues, or domestic conflict, did not feature within the reports.

The overall quality of all articles was assessed to be acceptable against Samaritans' guidelines. However, several problems with reporting were identified. A large proportion of articles failed to include contact details signposting readers to suicide prevention organizations (62, 46.3%) with an even larger number not including details of support organizations tailored for the nursing profession (129/131, 98.5%). Method of suicide was reported in over half of articles (70, 52.2%). Fourteen articles were highlighted as containing problematic language. This included romanticizing language, such as referring to ‘heroism’, or ‘selflessness’, sensationalist language, such as ‘suicide scandal’, and over‐simplification, where speculation about suicide triggers did not acknowledge the complexity of suicide. Finally, the majority of articles did not include messages related to suicide prevention (115/131, 87.8%), for example, encouraging individuals in distress to access support.

### Images

Most articles included images (116, 86.6%), with 41/116 (35.3%) containing three or more images, and two articles both containing 11 each. Most images were specific to the reported event; however, a few reports included stock images (images available from stock photography libraries). Over a quarter of reports (32/116, 27.6%) included occupation‐related images, such as the workplace of the individual who had died by suicide, or the individual in nursing uniform. Of COVID‐19‐related articles which included images (22/26, 84.6%), half (11/22, 50.0%) contained a pandemic‐related image, such as an individual wearing personal protective equipment, or a picture of COVID‐19 wards. Across all reports, images most commonly included were those of the individual who had died by suicide. Nearly, a quarter of online articles (18/80, 22.5%) contained videos. Video content varied considerably from instructions on how to contact suicide prevention organizations to inclusion of images of an individual's body on a stretcher. The image content of articles was largely assessed as acceptable (114/116, 98.3%) with a minority of articles (2/116, 1.7%) having a negative rating. These ratings were the result of images of the deceased's body on a stretcher, and a photograph of a ‘beauty spot’ associated with a suicide.

## DISCUSSION

We analysed the coverage of nurse and nursing student suicide by newspapers in the UK over a three‐and‐a‐half‐year period. This included reports published before and during the COVID‐19 pandemic. Overall, reporting was judged as acceptable. However, lack of adherence to media guidelines was still common. Occupation was frequently discussed within articles, at times being suggested as a factor contributing to suicide. Of articles published during the pandemic, links were often made between COVID‐19 and the suicide of nurses, with working on the frontline and fear of spreading COVID‐19 speculated as influential.

The findings of the present study expand on previous research using the same content analysis approach (Marzano *et al*. 2018; Marzano *et al*. 2022) by examining additional features of news reporting related to a specific occupation, in this context, nurses and associated students. Most articles reviewed included occupation‐related content, including stating profession (or nursing course) in the article headline, images of workplace or an individual in nursing uniform, and discussion of possible work or university‐related triggers for suicide. Inclusion of these features may increase the likelihood of nurses or students who read the content identifying with the individual featured in the article, which, if individuals are already vulnerable, may in turn increase the risk of contagion (e.g., Niederkrotenthaler *et al*. 2020). This is of concern given the known increased risk of suicide in nurses (Alderson *et al*. 2015; Davis *et al*. 2021; Windsor‐Shellard & Gunnell 2019).

The impact of media coverage of suicide on those bereaved (including colleagues) has been explored qualitatively (Gregory *et al*. 2020). Press intrusion, speculation, and inaccurate reporting were distressing to the bereaved. Media reports may be particularly difficult for nurses if their practices, profession, or workplace are publicly criticized. Inappropriate reporting of suicide may reinforce negative connotations surrounding nursing. This might include nurses being viewed as feminine, subservient, caring ‘*angels’* (Hoeve *et al*. 2014; Garcia & Qureshi 2022) who lack resilience. These connotations may lead to a negative professional self‐concept (Hoeve *et al*. 2014) having the potential to contribute to poor mental wellbeing, thus resulting in understaffing due to absence, poor staff recruitment, and difficulties in staff retention.

Although overall reporting was of acceptable quality, several problems in reporting were common. Method of suicide was often included within article text and headlines. This has been shown to be associated with increased suicide rates (Sinyor *et al*. 2018), possibly by increasing cognitive availability of a specific method, particularly for highly lethal or novel methods (e.g., Chen *et al*. 2013). Reporting of methods has also been shown to be distressing for those bereaved by the loss of individuals featured in news articles, including colleagues (Gregory *et al*. 2020).

Reporting of suicide among nurses may impact mental health nurses and students differently to nurses in other specialities. These clinicians are highly likely to be exposed to suicidal behaviour within the workplace (e.g., Takahashi *et al*. 2011; Windfuhr & Kapur 2011) and their training and experience may help them cope with difficult emotions experienced when reading media reports of suicide in nurses. On the other hand, occupational stress and cognitive availability of suicide methods within mental health nurses is already likely to be high due to workplace exposure. This might be in addition to previous or current exposure to suicidal behaviour in their personal lives as some mental health nurses may choose to enter the profession due to experiencing or knowing someone experiencing mental illness (Oates *et al*. 2018). Exposure to suicide is associated with suicide risk in the general population (e.g., Hill et al., 2020), and multiple exposure from personal, workplace and media‐related sources may contribute to increased risk. Lyra *et al*. (2021) note the paucity of research investigating possible links between exposure to suicide and suicidal thoughts and behaviours of professionals, including mental health nurses, and posit that such a correlation may exist. Research with crisis workers has shown exposure to personal, occupational, or colleague suicide was not associated with suicide ideation (Long *et al*. 2022). However, the impact of media attention to suicide was not explored and furthermore while the crisis workers worked in the context of mental health crises, they were not mental health nurses.

Mental health nurses may be especially sensitized to media reporting of suicide in nurses given that suicides of mental health patients often result in criticism of staff practice, including naming of staff by the media. Indeed, mental health professionals have been found to experience fear (Murphy *et al*. 2022) and distress (Sherba et al., 2019) related to negative publicity regarding suicides of patients. This may be increased if the suicide is of a student nurse or fellow professional, or if mental health nurses have patients on their caseload who are nurses or nursing students.

Many articles did not include contact details for support organizations, or messages relating to overcoming suicidal crises; for example, encouraging those in distress to seek support. The inclusion of signposting and prevention messages has been shown to influence help‐seeking and suicide reduction in traditional and new media (Niederkrotenthaler *et al*. 2010; Niederkrotenthaler *et al*. 2021). For example, the release and subsequent media attention related to music artist Logic's song “*1–800–273‐8255*” in the USA was followed by a very large increase in calls to a suicide prevention phone‐line and a 5.5% reduction in the national suicide rate, although this reduction was short‐lived (Niederkrotenthaler *et al*. 2021). Additionally, media narratives of hope and recovery have been shown to have a protective effect regarding suicide ideation (Niederkrotenthaler *et al*. 2022). Also, prevention‐framed media has been shown to be valued by bereaved individuals (Skehan *et al*. 2013).

In keeping with the analysis of UK reporting of suicide in relation to COVID‐19 using the same systematic method (Marzano *et al*. 2022), a small peak in reporting of suicides in nurses occurred at the beginning of the pandemic. However, it is not known whether this reflected an increase in suicides among nurses, or increased media attention to the issue. The present study showed that articles concerning suicides of nurses during this period contained strong links to the pandemic through headlines, images, and article content. In addition, the current study also showed that feelings of isolation and poor mental health were frequently reported as contributing to suicide, but with fear of infecting others and working on the frontline also being prominent in reports.

As far as we are aware, this is the first systematic analysis of UK news reporting of suicide in nursing staff. This is important as female nurses have an elevated risk of suicide (e.g., Alderson *et al*. 2015; Davis *et al*. 2021; Windsor‐Shellard & Gunnell 2019). A strength of the research includes the use of an established data extraction, coding, and code counting method which has been utilized successfully for exploration of news reporting of suicide prior to and during the COVID‐19 pandemic (Marzano *et al*. 2018; Marzano *et al*. 2022). However, the study included analysis of online and print newspaper reports provided by an external media monitoring company which monitors mainstream media outlets only. Therefore, articles from non‐traditional outlets, such as nursing‐specific journals and newsletters were not included. This is an avenue for future research as our preliminary exploration of related articles in these outlets has also identified similar problems in reporting. Analysis of other media outlets, for example, televised or news through social media platforms, would also be valuable, given the dominance of online sources for information‐seeking, but also their relevance for suicide contagion and prevention (Ortiz & Khin Khin 2018).

Educational interventions for journalists may encourage traditional and new‐media outlets to follow publishing guidance. Interventions should increase knowledge about areas which are poorly understood. This may include defining sensational or romanticized reporting (Machlin *et al*. 2012), reinforcing the possible impact of poor reporting on suicide, and highlighting occupational groups where reporting may be particularly impactful. These interventions should also target occupation‐centred media outlets, such as nursing journals and newsletters which may have greater reach to readers who are at increased risk.

To the authors' knowledge, research has not yet been undertaken which examines whether media reporting of suicide of individuals in a specific occupation has an impact on suicide rates within that occupation. Such evidence would provide a rationale for inclusion of occupation‐related guidance within existing guidelines.

## CONCLUSION

Responsible reporting of suicide is highlighted as a suicide prevention strategy worldwide. Although overall quality reporting of suicides of nurses in the UK appears to be reasonably acceptable, it could be improved. Consideration of how to report suicide among other occupational groups already at increased risk is also required. Supporting the media to sensitively and responsibly report suicides of nurses and nursing students may have a role in suicide prevention. It could also assist in staff recruitment and retention by helping to reduce negative connotations surrounding the nursing profession.

## RELEVANCE FOR CLINICAL PRACTICE

Future qualitative research should explore the impact on nurses or nursing students of reading articles related to suicide among their fellow professionals, including colleagues. Elements to investigate might include emotional and psychological responses, such as trauma responses, alongside occupational effects, such as desire to leave the profession. Furthermore, the impact of being named in the media in relation to a suicide should be studied. This may be particularly salient for mental health nurses, who could be named as an individual involved in the support of the nurse or student who has died by suicide as well as through their being a colleague. Findings from such research could inform the content of workplace training and postvention support for nurses and nursing students. For example, education on where to access support following media attention regarding the suicide of a nurse.

Mental health nurses may be well placed to deliver postvention support to nurses from other specialities who are affected by a colleague's suicide, due to their insights into the impact of suicide, and professional experience of providing bereavement support within their role. However, it should be noted that there is a lack of guidance regarding postvention tailored for healthcare professionals (Malik *et al*. 2021), especially regarding the suicide of a fellow nurse or nursing student. Mental health nurses should be consulted in the development of postvention guidance.

It has been suggested that media guidelines may contrast with the preferences of bereaved individuals (Gregory *et al*. 2020). For example, some people bereaved by suicide may wish for cause of death to be reported. Gregory *et al*. (2020) recommend that sensitive consultation by journalists with representatives of the bereaved may minimize distress. In the case of the suicide of a nurse or nursing student, journalist consultation with a senior nurse representative from the workplace or university may be appropriate. Developing a workplace infrastructure and protocol to respond to media attention may ensure colleagues or those affected are appropriately supported and may minimize harmful speculation about occupational factors contributing to suicide. This is particularly important given negative stereotypes about nursing often perpetuated by the media (Garcia & Qureshi 2022).

## Authorship statement

KH, MH, and KL were responsible for conceptualization and design of the study. SG and MH were involved in data acquisition, curation, and analysis, with interpretation by SG, KH, and KL. SG drafted the manuscript, with KL, KH, and MH involved in reviewing and editing. The final version was approved by all authors.

## Supporting information


**File S1** Template data extraction form.

## Data Availability

Research data are not shared.
