# Supplementary material for: News reporting of suicide in nurses: A content analysis study
Source: Int J Ment Health Nurs. 2022 Aug 25;31(6):1513–22. doi: 10.1111/inm.13057 (PMC9804535; doi:10.1111/inm.13057)
Supplement: Supplementary file 1 — File S1 Template data extraction form. [file INM-31-1513-s001.docx]

| **Template Data Extraction Form** | |
| --- | --- |
| **Article Characteristics** | |
| Newspaper Date |  |
| Newspaper Type |  |
| Media Source |  |
| Article Title |  |
| Article Format |  |
| Page Number If Print |  |
| CM2 If Print |  |
| Circulation If Print |  |
| Times Shared If Online |  |
| Public Comments Visible Online |  |
| **Heading Analysis** | |
| Clear Relation To Suicide |  |
| Tone |  |
| Method Mentioned |  |
| Focus On Mental Health |  |
| Focus On Prevention |  |
| Region Named |  |
| Occupation Named |  |
| Issues Named |  |
| COVID Mentioned |  |
| **Story Analysis** | |
| Region |  |
| Gender |  |
| Age |  |
| Name |  |
| Occupation |  |
| Event Type |  |
| Method |  |
| **Content Analysis** | |
| Online Element (e.g., social media quotes) |  |
| Paper Focus |  |
| Verdict If Inquest |  |
| History Of Health Conditions/Disability |  |
| Included Quotes |  |
| Workplace/University Named |  |
| Colleagues Named |  |
| Patients Named |  |
| Supporting Hcps Named |  |
| Linking Suicide To Other HCP Who Have Died By Suicide |  |
| Reports To Clustering Within Profession |  |
| Work Suggested As Contributory Factor |  |
| Work Suggested As Main Issue |  |
| COVID Strongly Linked (COVID Only) |  |
| COVID Link(S) |  |
| **Quality Of Reporting** | |
| Overall Article Rating |  |
| Signposting To Support Organisations e.g., Samaritans |  |
| Signposting To Support Targeted For Occupation |  |
| Method Stated In Article |  |
| Prevention Framed Messaging |  |
| Content Of Message |  |
| Language Problems (e.g., Romanticising/Sensationalist) |  |
| **Image Analysis** | |
| Number Of Photos |  |
| Photo Rating |  |
| Occupation Related Photo |  |
| COVID Related Photo |  |
| Image (s) Content (Per Photo) |  |
| Video Included |  |
| Video Content |  |
